# Barriers and enablers to the implementation of a perinatal care bundle: the Perinatal Excellence to Reduce Injury in Premature Birth project

**DOI:** 10.3389/fped.2026.1717781

**Published:** 2026-09-16

**Authors:** Hayley McBain, Sam Tuvey, Alessandra Glover Williams, Sally Hedge, Noshin Menzies, Sarah Bates, Karen Luyt, The PERIPrem Steering Group

**Affiliations:** 1 Health Innovation West of England, Bristol, United Kingdom; 2 Health Innovation South West, Exeter, United Kingdom; 3 Grange University Hospital, Cwmbran, Wales, United Kingdom; 4 Great Western Hospital NHS Foundation Trust, Swindon, United Kingdom; 5 Bristol Medical School, University of Bristol, Bristol, United Kingdom; 6 University Hospitals Bristol and Weston NHS Foundation Trust, Bristol, United Kingdom

**Keywords:** implementation, maternity care, perinatal, preterm birth, quality improvement

## Abstract

**Background:**

Preterm birth is one of the most significant causes of child mortality and morbidity internationally. Although we have seen an overall reduction in neonatal mortality since 1990 there remains significant variation across the world. PERIPrem, is a care bundle that brings together 11 low-cost best-practice interventions for mothers and preterm babies. Through a structured and supportive implementation approach, using primarily Quality Improvement (QI) methodology, implementation of these evidence-based interventions has increased but remains varied across participating sites.

**Methods:**

This was a qualitative exploration of the barriers and enablers to implementation, and the behavior change techniques used to encourage adherence to the bundle, using semi-structured interviews. The COM-B model for behavior change was used as a framework for the interviews and the Theoretical Domains Framework and Behavior Change Techniques Taxonomy to guide the analysis.

**Results:**

Across two timepoints, 47 interviews were conducted with 29 staff from maternity, obstetrics and neonatology. The data revealed that capability, opportunity, and motivation functioned as both barriers and enablers to the implementation of the bundle. The qualitative interviews revealed that implementation was achieved through improvements in perinatal team culture, improving knowledge and skills, having strong leadership and access to the right resources. At its core, implementation depended upon having a care bundle that was evidence-based and a supportive and credible team. Implementation did not come without its challenges; notwithstanding the COVID-19 pandemic and the effects this had on staff, challenges included the complexity and size of the bundle, limited dedicated time and resources to enhance implementation, and the impact of hierarchical perinatal structures. These findings have provided insights that will advance our understanding about the processes that underpin successful implementation of a complex perinatal regional care bundle.

**Discussion:**

Several factors interacted to enhance as well as hinder the implementation of this complex perinatal care bundle. Over time, adherence to the PERIPrem bundle was enabled by the application of theoretically informed behavior change techniques. To improve future delivery of complex care bundles, like PERIPrem, the processes and support provided to aid implementation should reflect on how these barriers can be overcome and the enablers enhanced.

## 1 Introduction

Globally 2.3 million children died in the first 28 days of life in 2022, 75% in the first week and 1 million within 24-hours (1). Although this is a significant reduction from 5 million babies in 1990, the decline has been slower than that of post-neonatal under-5 mortality and gains have reduced since 2010. Annual neonatal mortality rates vary significantly across the world, with West and Central Africa and South Asia experiencing the highest rates (1). It is now predicted that 64 countries will not meet the Sustainable Development Goal (SDG) target of reducing neonatal mortality rates to 12 deaths per 1,000 live births or fewer by 2030 unless urgent action is taken (1).

Preterm birth remains one of the leading causes of neonatal deaths internationally (2) and is also associated with severe injury and a predisposition to a range of motor and cognitive disabilities (3). To achieve the SDG target and reduce the burden of brain injury, novel treatments are currently being trialed for preterm babies and their mothers with promising results, but there are also existing low-cost evidence-based interventions that could be implemented with immediate effect (4). Care bundles have been widely used within the field of perinatal care to implement evidence-based interventions to address mortality and morbidity in preterm infants. In 2016, NHS England launched the first version of the Savings Babies’ Lives Care Bundle to improve antenatal and intrapartum care. Now in its third version, the bundle has grown from four to six elements and implementation in the UK has increased over time. The reduction in stillbirth rates in participating sites has also exceeded that reported nationally in the same period (5). The Safer Baby Bundle in Australia (6), took learning from the Saving Babies Lives Bundle, and when implemented in the state of Victoria led to a 21% reduction in stillbirth rates across participating maternity services in 2020–21 (7).

More recently, the Perinatal Excellence to Reduce Injury in Premature Birth (PERIPrem) project was launched in the UK. PERIPrem is a perinatal care bundle that brings together 11 evidence-based interventions that individually are associated with significant reductions in preterm neonatal mortality and morbidity (Table 1). To support implementation of the care bundle, PERIPrem uses Quality Improvement (QI) methodology, found to be effective in implementing other perinatal interventions (22, 23). Alongside this, teams are given individualized coaching support, regional learning events, backfill for two PERIPrem Leads (one Neonatal Nurse and one Midwife), an adaptable toolkit, and a personalized checklist known as the PERIPrem passport. Starting in 12 acute NHS Trusts in the South West and West of England, PERIPrem has now spread to Wales and other parts of the UK. Analysis has shown that taking this structured and supportive approach is associated with significant improvements in the delivery of the interventions individually, significantly increased the proportion of interventions that a mother and baby receive, and the proportion of mothers and babies who receive all interventions that they are eligible for (24). Further exploration is, however, needed to understand what explains variation in implementation of PERIPrem across sites and identify recommendations for future spread of the care bundle.

**Table 1:** Evidence-base for the PERIPrem care bundle.

Element	Description	Optimal timing	Evidence
Place of birth	Babies <27 ^+^ 0 weeks gestation (<28 ^+^ 0 weeks multiples) or <800 g who are born in a tertiary NICU	n/a	Extremely pre-term babies born in a non-tertiary unit are 2.3 times more likely to develop severe brain injury and 1.3 times more likely to die whether transported or not compared to controls (8)
Antenatal steroids	Mothers who give birth at <34 weeks gestation receive at least one dose of antenatal steroids	2 doses 12–24hrs apart, >24hr and <7days prior to birth	Reduces the risk of neonatal death by 31%, NEC by 50% and grade 3–4 IVH by 45% (9)
Magnesium sulphate	Mothers who give birth at <30 weeks gestation receive antenatal magnesium sulphate	>4hrs and <24hrs prior to birth	Reduces the risk of cerebral palsy by 32% (10)
Intrapartum antibiotics	Mothers who are in active labor at any point prior to delivery receive intrapartum antibiotics	At least 4hrs prior to birth	Reduces risk of neonatal GBS sepsis in GBS colonized women by 86% (11) Reduces the risk of delivering within 48hrs by 29% and within a week by 21% and abnormal neonatal cranial ultrasound by 19% (12)
Optimal cord management	Babies born at <34 weeks gestation have their cord clamped	At or after one minute of birth	Reduces mortality by 32% compared to early cord clamping (13)
Thermoregulation	Babies born at <34 weeks gestation have a normothermic temperature (36.5–37.5°c)	Within 1hr of admission to the neonatal unit	28% increase in mortality per 1°c decrease in body temperature (14) Moderate hypothermia associated with higher odds of IVH (OR 1.3) and death (OR 1.5) compared to a normothermic temperature (15)
Ventilation	Babies born at <34 weeks gestation who need invasive ventilation are given volume targeted ventilation in combination with synchronized ventilation as the primary mode of respiratory support	At birth	Reduces death or BPD by 27% and IVH (Grade 3–4) by 47% compared to PLV modes (16)
Caffeine	Babies born at <30 weeks gestation and/or <1,500 g receive caffeine therapy	Within the first 24hrs of life	The odds of death or BPD decrease by 19% (17). The odds of significant neurodevelopmental impairment decrease by 32% when caffeine is received within 2 days of birth (18)
Early breast milk	Babies born at <34 weeks gestation receive 1st MBM	Within the first 6hrs of life	Reduces the risk of NEC by 38% compared to formula (19)
Multi strain probiotics	Babies born at <32 weeks gestation and/or <1,500 g are started on multi strain probiotic	Within the first 24hrs of life	The odds of death are 44% less and the odds of developing NEC are between 45 and 69% less when receiving probiotics compared to a placebo (20)
Prophylactic hydrocortisone	Babies born at <28 weeks gestation are started on hydrocortisone	Within the first 24hrs of life	The odds of survival without BPD significantly increase by 45% and the odds of death before discharge reduce by 30% (21)

NEC, necrotizing enterocolitis; NICU, neonatal intensive care unit; MBM, maternal breast milk; IVH, intraventricular hemorrhage; GBS, group B streptococcal; OR, odds ratio; BPD, bronchopulmonary dysplasia; PLV, pressure-limited ventilation.

## 2 Materials and methods

### 2.1 Aims

The aims of this evaluation were to:

- understand the barriers and enablers associated with delivery of PERIPrem
- identify the behavior change techniques (BCTs) used to increase the uptake of a complex, perinatal care bundle.

### 2.2 Study design

This was a qualitative investigation, conducted alongside a quasi-experimental approach to understanding intervention effectiveness. The reporting and conduct of this study were guided by criteria for reporting qualitative research (COREQ) (25) (Supplementary File 1).

### 2.3 Setting

This study focuses on implementation of the PERIPrem bundle between July 2020 and June 2021. This was across a regional perinatal network of 12 acute NHS Trusts in the South West and West of England, UK with a total population of approximately five million people.

### 2.4 Participants

All eligible staff were invited to participate in the study. Those invited were either PERIPrem Leads (those backfilled), PERIPrem Champions (staff who led implementation of elements of the bundle) or PERIPrem regional QI coaches (based in the Health Innovation Network).

### 2.5 Data collection

Interviews were conducted virtually on two occasions, approximately 6 months apart; at the start of implementation and towards the end of the project. Those invited were provided with an information sheet about the study and gave verbal consent at the start of the interview. The interview schedule can be found in Supplementary File 2. Interviews were undertaken by ST (male), a member of the evaluation team who had undertaken the quantitative analysis of the PERIPrem impact data. ST holds a PhD in Sports and Exercise, with significant expertise in mixed-methods research and master's level training in qualitative techniques. The interviewer did not have prior relationships with the healthcare staff interviewed but worked alongside the QI coaches based within the Health Innovation Network, a regional organisation who were leading and funding the project's implementation.

### 2.6 Data analysis

All interviews were conducted via Microsoft Teams^©^ and then transcribed and anonymized in Otter AI^©^. Transcripts were not returned to participants for comment or correction; field notes were not taken. AI was not used to analyze the data.

A three-step framework and thematic analysis was used to analyze the data. The approach used evidence-based frameworks for behavior change, often found to be lacking in maternity care implementation research (26). The initial analysis was undertaken by HM (female), a Health Psychologist with significant experience of undertaking qualitative data collection and analysis. A second coder (ST) analysed 5 (11%) of the transcripts, and disagreements were discussed until consensus was reached.

- Using NVivo 12^©^ (27), framework analysis was used to deductively code the data into the domains from the Theoretical Domains Framework (TDF) for behavior change (28). These were then grouped by the three components of the COM-B model (29).
- Concurrently, the Behavior Change Technique Taxonomy version 1 (BCTTv1) (30) was used to identify the BCTs used within units to implement the bundle.
- Inductive thematic analysis was then conducted within TDF domains to create subdomains that described how that domain acted as a barrier or facilitator to implementation, and how the domain and subdomains interacted.
- Concurrently, each BCT was operationalized to describe the specific way in which that technique facilitated implementation of the bundle and what barriers or enables it was seeking to target, with examples.

The findings of the evaluation were taken to a PERIPrem regional webinar, with those interviewed in attendance. The findings were presented in line with the three study objectives, and the group were asked to reflect on whether these results aligned with their understanding and experiences, if we had misinterpreted any of the supporting quotes and themes, and if there were additional recommendations that should be developed. Minimal changes were made in response to this session.

### 2.7 Parent involvement

Parents with a lived experience of pre-term birth were members of the PERIPrem steering group, and as part of this group provided guidance and comment on the evaluation protocol (including the quasi-experimental study) and interpretation of findings. Parents also reviewed and provided comment on the interview schedules.

## 3 Results

A total of 47 interviews were conducted with 25 PERIPrem Leads and Champions, and four QI Coaches (100%) across 12 sites between November–December 2020 and May–June 2021. Participants included midwives (*n* = 9), neonatal nurses (*n* = 10), obstetricians (*n* = 4) and neonatologists (*n* = 2). The 2 waves of interviews did not consist of the same cohort due to staff changes. Successful implementation was influenced by interacting factors related to staff capability, opportunity and motivation. Domains and subdomains can be found in Supplementary File 3.

### 3.1 Capability

#### 3.1.1 Psychological and physical capability

Knowledge, skills and confidence [TDF: knowledge, skills, beliefs about capability] were closely linked and influenced participants’ ability to implement the PERIPrem bundle.

“If people don't know how [the bundle] can be achieved, they panic at it, so it is a lack of knowledge at the minute.”

Early interviews highlighted limited awareness of the bundle, its benefits for preterm babies specifically, and uncertainty around timing and delivery of interventions, particularly among more junior staff. This reduced confidence and acted as a barrier to implementation. An example of this was in the context of early breast milk:

“I speak to a lot of midwives, everyone has always known that breast milk is best, but I think that the implications of breast milk for the preterm infant probably weren't as widely known previously to PERIPrem.”

Although the PERIPrem brand brought a sense of identity, which in many ways enabled the elements to be viewed as a single entity, the qualitative interviews revealed that units did see the bundle as 11 individual elements. The size and complexity of the bundle proved challenging [TDF: memory, attention and decision processing], and participants described difficulties addressing all elements simultaneously, which meant it could be easily forgotten within an already complex and challenging environment.

“I think there are some elements which are simpler than others, potentially. So, giving steroids, giving magnesium sulphate, giving caffeine, they're kind of one-off events. And it's mostly about that decision making, they're kind of simpler. I think the more complicated, the more people involved in an element”

Staff capacity was further constrained by workload pressures, chronic staffing shortages and the COVID-19 pandemic, limiting attention and emotional resilience [TDF: memory, attention and decision processing].

“Everyone's resilience buckets are empty. So, their capacity to think about improving care is limited, because often it's just a matter of surviving and trying not to make mistakes. Rather than go ‘How can we get better?’”

To address a lack of knowledge, skills and confidence, PERIPrem Leads used the BCTs (i) instruction on how to perform the behavior, (ii) demonstration of the behavior (iii) self-monitoring of behavior and (iv) practice and rehearsal. Implementation relied on flexible educational approaches integrated into routine practice. PERIPrem Leads adapted education and communication strategies to be simple, targeted and timely (Table 2). Practical demonstrations, simulation, and opportunities for practice supported skill development and confidence.

**Table 2:** The techniques used to enhance knowledge, skills, and confidence.

Technique [BCT]	Example(s)
Staff were advised on how to perform each of the bundle interventions and QI methodology *[Instruction on how to perform the behavior]*	Instructions on how to perform QI and the bundle interventions were disseminated via a range of methods including the materials and resources made available through the PERIPrem project and events, locally developed guidelines, and the education and training given by the PERIPrem Leads.
Staff were shown how to perform interventions, either directly or indirectly *[Demonstration of the behavior]*	PERIPrem Leads and unit teams were shown examples of how their unit and others in the region had implemented elements of the bundle. These were presented during PERIPrem Share & Learn sessions, via the PERIPrem newsletter and regional events.
As part of QI, staff monitored their adherence to the bundle interventions *[self-monitoring of behavior]*	The PERIPrem team provided monthly personalized visualizations of their data over time to each of the units, and this information was then disseminated to unit staff via posters and training sessions. Staff also used the PERIPrem data collection tools to self-monitor. The data was used to solve problems (see Table 4), with data continually monitored as part of QI PDSA cycles.
Staff were prompted to rehearse performance of certain interventions *[practice and rehearsal]*	Simulations were used across units to enable staff to practice optimal cord management and thermoregulation. To increase staff skills, confidence, and habit formation in a safe environment.

QI, quality improvement; PDSA, plan, do, study, act.

To overcome the challenges with memory, attention and decision processing Leads used strategically placed nudges [BCT: prompts and cues], and broke the bundle into smaller, more manageable components to support adherence [BCT: graded tasks] (Table 3).

**Table 3:** Techniques to overcome barriers related to memory, attention, and decision processes.

Technique [BCT]	Example(s)
Environmental and social stimuli were introduced to the unit to prompt performance of the bundle interventions *[Prompts and cues]*	• PERIPrem passports, infographics and stickers were used and PERIPrem display boards were introduced. • Bundle interventions were added to pre-existing checklists (e.g., WHO-SCC (32)). • Magnets were used on the delivery suite board to indicate a baby of <34 weeks gestation. • PERIPrem Leads and PERIPrem Champions were present on the ward reminding and supporting staff.
PERIPrem Leads described breaking the bundle down into interventions that were easier to introduce and those that were more complex *[graded task]*	Units decided on which interventions to address by assessing each element according to their current level of delivery, its perceived complexity and applicability in their context.

WHO-SCC, World Health Organization Safe Childbirth Checklist.

### 3.2 Motivation

#### 3.2.1 Reflective motivation

Beliefs about the benefits [TDF: beliefs about consequences] of the bundle and the strength of the underpinning evidence were key motivators. Once staff understood the impact of individual interventions and the value of delivering them as a bundle, buy-in was generally high. This is exemplified in a quote about the evidence for delayed cord clamping:

“The one that has stuck in my head since I read it is about the delayed cord clamping reducing mortality by a third, which just boggles my mind how that is even possible. But because it is when I've then told that to other people, they've had the same reaction I had. And suddenly it's then sparked that ‘okay, this is a good thing, we should do this”

Beyond belief in the individual interventions, participants described PERIPrem as a trusted and recognizable program. Credibility was enhanced through endorsement from the UK, South West Neonatal Network and the Neonatal Nurse Association [TDF: social influence], input from respected clinicians [BCT: credible source; social support], high-quality resources [TDF: environmental context and resources], strong evidence [TDF: beliefs about consequences] and ongoing coaching support [BCT: credible source; social support].

“I think it's been good to have the national backing of bodies like BAPM [British Association of Perinatal Medicine]. And to know that this is being backed by our governing body if you like, that's been helpful.”

A shared purpose [TDF: goal] to improve outcomes for preterm babies motivated multidisciplinary collaboration. Parent stories were described as particularly powerful, helping staff connect their actions to longer-term outcomes for babies and families.

“We have a motivated team I think of people that are interested in doing the best by these women and babies.”

Resistance was reported for some bundle elements, particularly among more senior clinicians. This was attributed to concerns about evidence strength, perceived risks in specific patient groups, and greater risk aversion [TDF: beliefs about consequences]. This led to elements not being delivered or a lengthier process to gain consensus on elements of the bundle.

“I think, in general, a lot of healthcare workers of that level, so consultants, this is across obstetricians and neonatologists are often relatively risk averse when it comes to change, particularly if they don't have problems at the moment. Because obviously you don't want to introduce something and then everything gets worse, because it's lives, we're dealing with, and in this case, very, very little lives.”

To enhance reflective motivation, the project team and PERIPrem Leads used a range of techniques (Table 4). The benefits of implementing the bundle (BCT: information about consequences) were at the core of the PERIPrem approach. Teams regularly reviewed their data on implementation of the bundle elements and addressed barriers [BCTs: self-monitoring and problem solving] and conducted tests of change [BCT: behavioral experiments].

**Table 4:** Techniques used to address reflective and automatic motivation.

Technique [BCT]	Example(s)
Written, verbal and visual information was provided to PERIPrem Leads and unit staff about the health benefits of each element of the bundle *[Information about health consequences]*	All PERIPrem resources and events stressed the evidence base for each of the interventions, this information then filtered down to unit staff through locally developed guidelines, education and training events, resources, and materials.
Units used their data to analyze the factors influencing the behavior and generate and select strategies to overcome the barriers and/or increase the enablers *[problem solving]*	As part of PDSA cycles, PERIPrem Leads worked with the unit staff to review past data and understand barriers and enablers. In some cases, this was facilitated by full case reviews of all affected babies, more achievable in smaller units with fewer babies meeting the PERIPrem criteria.
PERIPrem Leads identified and tested hypotheses about implementing the PERIPrem bundle by collecting and interpreting data *[behavioral experiments]*	As QI was at the core of the PERIPrem approach, PERIPrem Leads were continuously conducting tests of change across the bundle elements, using data to drive their decision making.
Positive reinforcement was provided when effort and progress was made towards performing an intervention *[social reward]*	The PERIPrem team and PERIPrem Leads made significant effort to celebrate the accomplishments of the units and staff throughout the project.

QI, quality improvement; PDSA, plan, do, study, act.

#### 3.2.2 Automatic motivation

Celebrating success reinforced motivation (BCT: social reward) and encouraged future implementation, fostering a sense of pride within teams (Table 4).

“And I think some of it is about shared ownership. So, thanking them and making them feel like this isn't my work, like the project is my work, but the outcomes are their work. So, a lot of it has been about making sure they feel appreciated. And then I think they're more engaged into the project.”

### 3.3 Opportunity

#### 3.3.1 Physical opportunity

Participants reported widespread use of PERIPrem resources [TDF: environmental context and resources] and described adapting materials to local contexts, with infographics acting as prompts and communication tools. The implementation toolkit was used to support staff education, communication and delivery of bundle elements. Many participants identified these resources as an important facilitator of implementation. Use of PERIPrem passports varied across Trusts, with more consistent uptake in units undertaking detailed case reviews.

“all the posters that have come up for each of the different elements. I think they're so nicely and clearly written. They're not too wordy, and we've displayed them, we've got a PERIPrem board. So, it just simply tells you the key facts, simple things that people can actually understand. And actually, I did a training session with one of my registrars last night and she said ‘this is really simple stuff, but it just makes such a difference.’ And it's well presented. So, I think probably taking that back to the PERIPrem team that designed the posters that support each of the bundle elements, that's been really useful, because they're good training materials.”

The £500 implementation grant [TDF: environmental context and resource] enabled Trusts to purchase equipment essential for delivery of some interventions. Where equipment was unavailable, implementation was hindered, particularly for optimal cord management. Although Leads were also funded to protect their time on the project, in reality this was a challenge and hindered progress in some Trusts.

“And with ridiculous workload, it stops you doing it. It doesn't hit the top 10 of things I have to do in a day. It never actually gets in that top list.”

The COVID-19 pandemic influenced in both positive and negative ways: while pivoting to virtual events increased access for remote units; education, simulation, networking and staffing capacity were significantly constrained.

“So [COVID-19] absolutely knackered it, but it's also added loads to it. So, it's a bit of a tricky one. So, of course, the ideal situation would be that we wouldn't have any physical restrictions on where we could be and who we could sit next to and talk and bring people into a room because obviously, the human contact element is really important in establishing relationships with people and training. However, that said, I feel like we have a really strong library of training and resources that we wouldn't have had had we not had to do things virtually”

Unit size also influenced implementation [TDF: environmental context and resource]. Smaller units found training and communication easier and were able to undertake detailed case reviews, but fewer eligible cases limited opportunities to build routine and confidence. Larger units faced greater coordination challenges but had more frequent opportunities to practice bundle delivery.

“Yeah. Yeah. So [having fewer eligible babies] in itself, I think is quite a challenge because you're reminding people constantly ‘remember PERIPrem. I know we've had no babies, but we're still playing this game, and we're in it for the long haul, so please don't forget it.’”

To optimize the benefits of the physical environment, PERIPrem Leads described adding equipment and materials to the physical environment in order to facilitate performance of bundle elements [BCT: adding objective to the physical environment] (Table 5). These resources and equipment were in some cases funded through the PERIPrem project, but in many cases were innovations developed by the teams themselves.

**Table 5:** Technique used to enhance physical opportunity.

Technique [BCT]	Example(s)
PERIPrem Leads described adding equipment and materials to the physical environment to facilitate performance of the interventions *[adding objective to the physical environment]*	• Optimizing place of birth – creating a list of telephone numbers for the regional neonatal and obstetrics units and securing funding for quantitative fFN across the region thanks to Hologic^©^ • EBM – creating breast milk packs contain 1 mL syringes, date stickers, a leaflet for mother and bonding comforters like MIniboos • Optimizing place of birth and steroids – introduction of the QUiPP app; a decision support tool that uses medical history, quantitative fFN and cervical length to give an individualized score for the risk of having a spontaneous preterm delivery • All intervention – grab boxes, containing vital equipment and information

fFn, fetal fibronectin; EBM, early breast milk.

#### 3.3.2 Social opportunity

Participants frequently described the role of the PERIPrem Lead as central to implementation [TDF: social influence]. Successful implementation was associated with Leads who were enthusiastic, credible and able to engage colleagues across professional groups. Sites reporting greater implementation success often described stable leadership and dedicated implementation capacity, whereas turnover of Leads or limited protected time were perceived barriers.

“the really, really successful units were the ones who had massively enthusiastic people come forward wanting to be the PERIPrem Leads. So absolutely the number one ingredient for success is people; they have to be the right people. And the units where it took a lot longer for them to engage as well, was where we had to change those people several times, because of problems with availability or engagement. Where the units where people came forward first, remained really enthusiastic and keen for it has been the ones that have done really well. And you can absolutely see that units where they haven't got a strong maternity lead. That's been where they've really struggled. And they specifically struggled in the perinatal areas so deferred cord clamping, maternal breast milk, where you have to work together to fix it, you can't fix it in isolation. And that was definitely a strong theme was it was all about the people who were in that perinatal PERIPrem team, that was key.”

Beyond the Lead, the use of PERIPrem Champions [TDF: social influence] to take ownership of specific interventions helped distribute workload and increase engagement across teams. Senior leadership support at divisional level also enabled implementation by legitimizing change and, in some cases, protecting time. Although wider Trust-level engagement varied due to pandemic pressures, the absence of resistance was generally interpreted as tacit support.

“I think with individual consultants taking a lead on a certain area, so with delayed cord clamping, for example, one of our pediatric consultants not particularly involved in PERIPrem took that forward as an individual project. And so, having someone drive that individual project was really good, because they weren't kind of distracted by the other nine, they were just doing that thing themselves. And so that that worked quite well.”

Units with an established quality-improvement culture [TDF: social influence] reported smoother implementation, supported by access to QI expertise and coaching [TDF: social influence and credible source]. Embracing QI and having a change mindset was important not only at an individual PERIPrem Lead level but also at a team and Trust level.

“So, I think first thing I would note is that the willingness of the unit to embrace QI really correlated with how available they were to do the QI. So, I think units like [hospital X] who have fully embraced it, the kind of training and the coaching on offer, kind of really took advantage of what was on offer, and their kind of enthusiasm for it correlates with how much they took from the process, if that makes sense. Whereas a team like [hospital Y] who were very difficult to engage, were also reluctant to engage in the QI coaching, if that makes sense.”

Participants consistently described PERIPrem as a catalyst for stronger multidisciplinary collaboration [TDF: social influence] between maternity, obstetric and neonatal teams. Joint education sessions, co-produced guidelines and shared improvement activities were perceived to increase communication across professional groups and foster a more integrated perinatal team identity. Participants reported greater willingness to challenge practice and discuss care openly in settings where teamwork was strongest, although hierarchical structures remained a barrier in some units.

“there has been significantly more collaborative working, pediatricians scrubbing in at the table, taking responsibility for the risk of resus at the bedside or on the theatre table, which then takes the pressure off the obstetrician, the obstetrician doesn't have to make a judgement call on is this baby okay to have delayed cord clamping for a minute or not. And actually, that's worked really well. Everyone's engaged. It's good. Again, it's good collaborative working.”

To enhance the social opportunities that aided implementation of the bundle elements the PERIPrem project team and PERIPrem Leads provided extensive expertise and support [BCT: credible source and social support]. The Share & Learn sessions, newsletters and events, idea sharing and collective learning [BCT: social comparison] provided opportunities to share data and learn from other trusts (Table 6). The support offered by coaches was flexible and tailored to the needs of the individual units, acting as a reminder, a source of information and someone who was able to build relationships and galvanize teams into action. Co-production approaches, supported by both PERIPrem Leads and QI Coaches, fostered ownership, engagement and sustainability [TDF: social professional role and identity].

**Table 6:** Techniques used to improve social opportunities.

Technique [BCT]	Example(s)
PERIPrem Leads spoke about the encouragement and advice given by key members of the PERIPrem team *[social support]*	PERIPrem Leads were supported by the PERIPrem team and in particular QI coaches, via PERIPrem events and coaching support.
A range of verbal and visual communication was provided by credible people in favor of the PERIPrem approach *[credible source]*	The PERIPrem team and QI coaches, accompanied by specialists in perinatal care endorsed the bundle and evidence base throughout the project and particularly during the Share & Learn sessions. The bundle was also endorsed by the SW ODN and NNA.
PERIPrem Leads and QI coaches described the benefits of comparing practice, performance and learning between units *[social comparison]*	The PERIPrem newsletters, Share & Learn sessions and celebratory events provided units with the opportunity to share their current practice and data. This allowed units to compare themselves to other similar units within the region and share learning.

QI, quality improvement; SW ODN, South West Operational Delivery Network, NNA, Neonatal Nurse Association.

“It's about making people feel involved, isn't it? So, if they don't feel involved with something, they're much less partial to being so engaged. So, I've definitely put things out for people to come forward with their ideas on the bundle, and if they have any, and how it can be improved, or what they'd like to see or what can happen.”

## 4 Discussion

This study explored the barriers and enablers to implementing the PERIPrem care bundle within a regional perinatal network, using evidence-based theoretical frameworks for behavior change. The findings demonstrate that successful implementation was influenced by interacting factors relating to capability, opportunity and motivation. Key enablers included improved knowledge and skills, belief in the evidence underpinning the bundle elements, dedicated implementation leadership, multidisciplinary collaboration, access to practical implementation resources and quality improvement support. Conversely, implementation was challenged by the complexity of the bundle, competing clinical pressures, limited protected time, hierarchical professional cultures and the disruption associated with the COVID-19 pandemic. These findings reflect the organizational and cultural mechanisms that have been found to underpin safe maternity care (31).

The importance of having the skills and knowledge to implement the bundle elements and believe they will have a positive impact is consistent with previous implementation research demonstrating that healthcare professionals are more likely to adopt new practices when they understand both how to perform them and why they are beneficial (32, 33). Education, simulation training and local feedback mechanisms appeared to improve capability, whilst reinforcing the evidence base and sharing positive parent stories strengthened staff motivation.

Consistent with previous quality improvement and maternity safety research, implementation was facilitated by dedicated leadership, protected time for improvement activity, a team culture that embraced continuous learning and change, and organizational support (31, 34, 35). Participants emphasized the importance of protected time and implementation leads who were able to coordinate activity across professional groups. The findings also suggest that the complexity of large perinatal care bundles may require implementation responsibilities to be distributed across multiple champions in obstetrics, midwifery and neonatology, rather than relying on a single individual. PDSA cycles have been linked with increased adherence to care bundles (36) and PERIPrem Leads did indicate that behavioral experiments were taking place, alongside the monitoring of data for improvement, problem solving and action plans. Practical implementation resources, together with coaching and performance feedback, further supported the adoption of evidence-based practice in this complex and stressful environment.

A particularly important finding was the role of multidisciplinary collaboration and perinatal team culture. Participants described how PERIPrem encouraged greater integration between maternity, obstetric and neonatal services through shared goals, education and improvement activities. These findings align with evidence highlighting communication, teamwork and psychological safety as critical contributors to patient safety in maternity services. Communication failures and poor team functioning have been estimated to be the root cause of 72% and 55% of sentinel events in obstetrics respectively (37) and one of the key causes is hierarchical relationships in maternity services, and the fear of repercussions from colleagues to voice safety concerns (38). The PERIPrem project has shown that an existing positive perinatal team culture can enable delivery of a perinatal care bundle, but this needs to be fostered and developed. Conversely, hierarchical professional structures were identified as barriers to implementation, suggesting that future programs should consider strategies to foster collaboration across professional boundaries.

The COVID-19 pandemic influenced implementation in both positive and negative ways. Whilst virtual delivery increased access to training, coaching and regional learning opportunities, participants reported reduced opportunities for face-to-face networking and relationship building. Pandemic-related workforce pressures also reduced capacity for QI activity and contributed to staff fatigue. These findings are consistent with broader reports describing both the benefits and challenges of delivering QI initiatives during the pandemic (39).

These findings suggest that successful implementation was facilitated by a combination of evidence-based interventions, enhanced staff knowledge and skills, dedicated leadership, multidisciplinary collaboration and access to appropriate implementation resources. Challenges included bundle complexity, limited capacity for improvement activity, professional hierarchies and the ongoing effects of the COVID-19 pandemic. Future work should focus on understanding how implementation strategies can be tailored to different settings and sustain long-term adherence to complex perinatal care bundles.

## 5 Strengths and limitations

This study has several strengths. First, it is one of the first theoretically informed qualitative evaluations of the implementation of a perinatal care bundle. The study was guided by evidence-based frameworks from implementation science and behavior change research, providing a robust and transparent approach to understanding implementation processes and linking barriers and enablers to behavior change strategies. Second, data were collected across 12 NHS Trusts and included participants from maternity, obstetric and neonatal services with a range of professional backgrounds. The inclusion of interviews at two time points enabled exploration of implementation experiences throughout the course of the program rather than at a single point in time. Third, the study achieved high participant engagement, with all invited project leads, champions and QI coaches participating. The use of theory-based analysis, dual coding of a sample of transcripts, and discussion of coding discrepancies enhanced analytical rigor and credibility.

Several limitations should however also be acknowledged. Because the interviewer and analysts were employed by the organizations responsible for implementing and evaluating PERIPrem, there is a risk of interviewer and interpretative bias. Participants may have been less willing to express critical views, and researchers may have unintentionally interpreted findings more favorably. Although steps were taken to enhance analytic rigor, this may have influenced the credibility and confirmability of the findings. In addition, participant checking was undertaken through a regional webinar, which represents a relatively limited form of respondent validation. Attendance was voluntary, feedback was provided in a group setting, and participants reviewed summary findings rather than transcripts or detailed analyses. Consequently, the process provided broad confirmation of the findings but may not have identified all areas of disagreement or alternative interpretations.

The findings are also presented at an aggregate level and do not differentiate between participant roles, levels of seniority, organizations or interview waves. This approach was consistent with the study objectives and helped protect participant anonymity given the small numbers within some groups. However, it has limited opportunities to explore variation in experiences across professional groups, organizational contexts and time. In particular, despite the longitudinal design, the analysis did not formally compare findings across the two interview waves and therefore cannot comment on how perceptions or experiences evolved during implementation. Future longitudinal implementation studies should consider incorporating planned comparative analyses across participant groups and time points.

Finally, interviews were conducted primarily with PERIPrem Leads, Champions and QI coaches who were directly involved in program implementation. The perspectives of frontline staff responsible for delivering the individual bundle elements were not explored and may have identified additional barriers and enablers. Furthermore, while the evaluation examined implementation of the bundle as a whole, individual interventions may have been associated with distinct implementation challenges that warrant further investigation.

## 6 Implications for research and clinical practice

This study highlights several considerations for evaluating and implementing complex perinatal care bundles such as PERIPrem in routine clinical practice.

Future research should explore how implementation experiences differ across professional groups, organizations and stages of implementation, as well as identify which implementation strategies have the greatest influence on long-term adherence to individual care-bundle elements. Further investigation of how multidisciplinary culture and leadership influence implementation outcomes may also help optimize the spread and sustainability of complex perinatal interventions.

Successful implementation requires more than simply disseminating evidence-based interventions. Given the number and complexity of the PERIPrem bundle elements, implementation strategies should help staff integrate interventions into existing clinical workflows. Practical tools such as patient passports (checklists) with structured case reviews, visual reminders and delivery suite prompts appear particularly important for reducing cognitive burden and supporting reliable delivery of multiple interventions during time-critical clinical situations.

Implementation of perinatal care bundles requires coordinated action across maternity, obstetric and neonatal teams. Organizations should therefore prioritize opportunities for multidisciplinary learning, joint guideline development, simulation training and shared improvement activities. These approaches may help strengthen perinatal team culture, improve communication across professional groups and reduce the impact of hierarchical barriers that can impede implementation.

The findings suggest that implementation is facilitated by dedicated leadership and protected time. Future programs should ensure that implementation leads have sufficient capacity to undertake improvement activities and that responsibilities are distributed across champions responsible for individual bundle components. This may be particularly important for larger bundles, such as PERIPrem, where implementation demands are substantial and need to be sustained over time.

Local adaptation should be considered an important feature of implementation rather than a deviation from protocol. Participants described adapting educational materials, resources and implementation strategies to suit local circumstances while maintaining fidelity to the clinical components of the bundle. Supporting this balance between consistency and local flexibility may enhance acceptability and sustainability.

Finally, programs aiming to improve outcomes for pre-term infants should consider combining evidence-based clinical interventions with structured implementation support, including improvement coaching, opportunities for peer learning, regular feedback of performance data and mechanisms for sharing learning across organizations. These approaches may help address common barriers related to capability, opportunity and motivation and support sustained improvement in bundle adherence.

## Data Availability

The datasets presented in this article are not readily available because data retention policies mean that the raw data from these interviews has been destroyed and no longer available. Requests to access the datasets should be directed to HM hayley.mcbain1@nhs.net.

## PERIPrem Steering Group

Teddy Baker, Tamsin Boon, Bernadette Chubb, Joanna Garrett, Pippa Griew, Kay Haughton, Tracy Jolly, Tracey Kay, Matthew Larkin, Mary Leighton, Rebecca Lemin, Wisam Muhsen, Ann Remmers, Adam Smith-Collins, Charlotte Sullivan, Rachel Tompkins, Emma Treloar, Amie Turner, Leanna Wakely, Ellie Wetz, Rebecca Whitting

## Supplementary material

SUPPLEMENTARY FILE 1 Interview schedule.

SUPPLEMENTARY FILE 2 COREQ checklist.

SUPPLEMENTARY FILE 3 Barriers and enablers to implementation (COM-B by TDF).

## References

[1] Hug L Alexander M You D Alkema L. National, regional, and global levels and trends in neonatal mortality between 1990 and 2017, with scenario-based projections to 2030: a systematic analysis. Lancet Glob Health. (2019) 7(6):e710–20. 10.1016/S2214-109X(19)30163-9 31097275 PMC6527519

[2] Williams T Sleap V Stoianova S Rossouw G Cook N Odd D. National Child Mortality Database Programme (Second Annual Report): Data from April 2019 to March 2020. London: Healthcare Quality Improvement Partnership (2021). Available online at: https://www.ncmd.info/wp-content/uploads/2021/06/NCMD_2nd_Annual_Report_June-2021_web-FINAL.pdf (Accessed November 10, 2025).

[3] Gale C Statnikov Y Jawad S Uthaya S Modi N; Brain Injuries Expert Working Group. Neonatal brain injuries in England: population-based incidence derived from routinely recorded clinical data held in the national neonatal research database. Arch Dis Child Fetal Neonatal Ed. (2018) 103:F301–6. 10.1136/archdischild-2017-313707 29180541 PMC6047140

[4] Huynh LT Tran HT Vanhaesebrouck S. Review of low-cost interventions for reducing neonatal mortality and morbidity in low- and middle-income countries. Acta Paediatr. (2025) 114(9):2124–32. 10.1111/apa.70133 40364732

[5] Widdows K Roberts SA Camacho EM Heazell AE. Stillbirth rates, service outcomes and costs of implementing NHS England’s saving babies’ lives care bundle in maternity units in England: a cohort study. PLoS One. (2021) 16(4):e0250150. 10.1371/journal.pone.0250150 33872334 PMC8055032

[6] Andrews CJ Ellwood DA Gordon A Middleton P Homer CSE Wallace EM. Stillbirth in Australia 2: working together to reduce stillbirth in Australia: the safer baby bundle initiative. Women Birth. (2020) 33(6):514–9. 10.1016/j.wombi.2020.09.006 33092700

[7] Stillbirth Centre of Research Excellence. The Safer Baby Bundle (2025). Available online at: https://www.stillbirthcre.org.au/about-us/our-work/the-safer-baby-bundle/ (Accessed July 2026).

[8] Helenius K Longford N Lehtonen L Modi N Gale C; Neonatal Data analysis Unit and UK Neonatal Collaboration. Association of early postnatal transfer and birth outside a tertiary hospital with mortality and severe brain injury in extremely preterm infants: observational cohort study with propensity score matching. Br Med J. (2019) 367:l5678. 10.1136/bmj.l5678 PMC6812621 31619384

[9] Roberts D Brown J Medley N Dalziel SR. Antenatal corticosteroids for accelerating fetal lung maturation for women at risk of preterm birth. Cochrane Database Syst Rev. (2017) 3(3):CD004454. 10.1002/14651858.CD004454.pub2 28321847 PMC6464568

[10] Doyle LW Crowther CA Middleton P Marret S. Magnesium sulphate for women at risk of preterm birth for neuroprotection of the fetus. Cochrane Database Syst Rev. (2009) 5(5):CD004661. 10.1002/14651858.CD004661.pub2 17636771

[11] Fairlie T Zell ER Schrag S. Effectiveness of intrapartum antibiotic prophylaxis for prevention of early-onset group B streptococcal disease. Obstet Gynecol. (2013) 121(3):570–7. 10.1097/AOG.0b013e318280d4f6 23635620

[12] Kenyon S Boulvain M Neilson JP. Antibiotics for preterm rupture of membranes. Cochrane Database Syst Rev. (2003) (2):CD001058. 10.1002/14651858.CD001058 12804398

[13] Fogarty M Osborn DA Askie L Seidler AL Hunter K Lui K. Delayed vs early umbilical cord clamping for preterm infants: a systematic review and meta-analysis. Am J Obstet Gynecol. (2018) 218(1):1–18. 10.1016/j.ajog.2017.10.231 29097178

[14] Laptook AR Salhab W Bhaskar B; Neonatal Research Network. Admission temperature of low birth weight infants: predictors and associated morbidities. Pediatrics. (2007) 119(3):e643–9. 10.1542/peds.2006-0943 17296783

[15] Miller SS Lee HC Gould JB. Hypothermia in very low birth weight infants: distribution, risk factors and outcomes. J Perinatol. (2011) 31(Suppl 1):S49–56. 10.1038/jp.2010.177 21448204

[16] Klingenberg C Wheeler KI McCallion N Morley CJ Davis PG. Volume-targeted versus pressure-limited ventilation in neonates. Cochrane Database Syst Rev. (2017) 10(10):CD003666. 10.1002/14651858.CD003666.pub4 29039883 PMC6485452

[17] Lodha A Seshia M McMillan DD Barrington K Yang J Lee SK. Association of early caffeine administration and neonatal outcomes in very preterm neonates. JAMA Pediatr. (2015) 169(1):33–8. 10.1001/jamapediatrics.2014.2223 25402629

[18] Lodha A Entz R Synnes A Creighton D Yusuf K Lapointe A. Early caffeine administration and neurodevelopmental outcomes in preterm infants. Pediatrics. (2019) 143(1):e20181348. 10.1542/peds.2018-1348 30518670

[19] Altobelli E Angeletti PM Verrotti A Petrocelli R. The impact of human milk on necrotizing enterocolitis: a systematic review and meta-analysis. Nutrients. (2020) 12(5):1322. 10.3390/nu12051322 32384652 PMC7284425

[20] Morgan RL Preidis GA Kashyap PC Weizman AV Sadeghirad B Chang Y. Probiotics reduce mortality and morbidity in preterm, low-birth-weight infants: a systematic review and network meta-analysis of randomized trials. Gastroenterology. (2020) 159(2):467–80. 10.1053/j.gastro.2020.05.096 32592699 PMC8014956

[21] Shaffer ML Baud O Lacaze-Masmonteil T Peltoniemi OM Bonsante F Watterberg KL. Effect of prophylaxis for early adrenal insufficiency using low-dose hydrocortisone in very preterm infants: an individual patient data meta-analysis. J Pediatr. (2019) 207:136–42. 10.1016/j.jpeds.2018.10.004 30416014

[22] Davis JW Odd D Jary S Luyt K. The impact of a sepsis quality improvement project on neurodisability rates in very low birthweight infants. Arch Dis Child Fetal Neonatal Ed. (2016) 101(6):F562–4. 10.1136/archdischild-2015-309804 27539482

[23] Edwards HB Redaniel MT Sillero-Rejon C Margelyte R Peters TJ Tilling K. National PReCePT Programme: a before-and-after evaluation of the implementation of a national quality improvement programme to increase the uptake of magnesium sulfate in preterm deliveries. Arch Dis Child Fetal Neonatal Ed. (2023) 108(4):342–7. 10.1136/archdischild-2022-324579 36617442 PMC10314002

[24] Glover Williams A Tuvey S McBain H Menzies N Hedge S Bates S. Perinatal excellence to reduce injury in preterm birth (PERIPrem) through quality improvement. BMJ Open Qual. (2022) 11(3):e001904. 10.1136/bmjoq-2022-001904 35944934 PMC9367190

[25] Tong A Sainsbury P Craig J. Consolidated criteria for reporting qualitative research (COREQ): a 32-item checklist for interviews and focus groups. Int J Qual Health Care. (2007) 19(6):349–57. 10.1093/intqhc/mzm042 17872937

[26] Dadich A Piper A Coates D. Implementation science in maternity care: a scoping review. Implement Sci. (2021) 16(1):16. 10.1186/s13012-021-01083-6 33541371 PMC7860184

[27] QSR International Pty Ltd. NVivo [computer software]. Melbourne: QSR International Pty Ltd (2020). Available online at: https://www.qsrinternational.com/nvivo-qualitative-data-analysis-software/home (Accessed August 2021)

[28] Cane J O'Connor D Michie S. Validation of the theoretical domains framework for use in behaviour change and implementation research. Implement Sci. (2012) 7:37. 10.1186/1748-5908-7-37 22530986 PMC3483008

[29] Michie S Van Stralen MM West R. The behaviour change wheel: a new method for characterising and designing behaviour change interventions. Implement Sci. (2011) 6:42. 10.1186/1748-5908-6-42 21513547 PMC3096582

[30] Michie S Richardson M Johnston M Abraham C Francis J Hardeman W. The behavior change technique taxonomy (v1) of 93 hierarchically clustered techniques: building an international consensus for the reporting of behavior change interventions. Ann Behav Med. (2013) 46(1):81–95. 10.1007/s12160-013-9486-6 23512568

[31] Liberati EG Tarrant C Willars J Draycott T Winter C Chew S. How to be a very safe maternity unit: an ethnographic study. Soc Sci Med. (2019) 223:64–72. 10.1016/j.socscimed.2019.01.035 30710763 PMC6391593

[32] Patey AM Islam R Francis JJ Bryson GL Grimshaw JM; Canada PRIME Plus Team. Anesthesiologists’ and surgeons’ perceptions about routine pre-operative testing in low-risk patients: application of the theoretical domains framework (TDF). Implement Sci. (2012) 7(1):52. 10.1186/1748-5908-7-52 22682612 PMC3522997

[33] Cox NS Oliveira CC Lahham A Holland AE. Pulmonary rehabilitation referral and participation are commonly influenced by environment, knowledge, and beliefs about consequences: a systematic review using the theoretical domains framework. J Physiother. (2017) 63(2):84–93. 10.1016/j.jphys.2017.02.002 28433238

[34] Stewart GL Manges KM Ward MM. Empowering sustained patient safety: the benefits of combining top-down and bottom-up approaches. J Nurs Care Qual. (2015) 30(3):240–6. 10.1097/NCQ.0000000000000103 25479238

[35] Drew JR Pandit M. Why healthcare leadership should embrace quality improvement. Br Med J. (2020) 368:m872. 10.1136/bmj.m872 PMC7190272 32234838

[36] Gilhooly D Green SA McCann C Black N Moonesinghe SR. Barriers and facilitators to the successful development, implementation and evaluation of care bundles in acute care in hospital: a scoping review. Implement Sci. (2019) 14:47. 10.1186/s13012-019-0894-2 31060625 PMC6501296

[37] The Joint Commission. Preventing infant death and injury during delivery. Oakbrook Terrace (IL): The Joint Commission (2004). Available online at: http://www.jointcommission.org/-/media/tjc/documents/resources/patient-safety-topics/sentinel-event/sea_30.pdf (Accessed July 2026)

[38] Elliott-Mainwaring H. How do power and hierarchy influence staff safety in maternity services? Br J Midwifery. (2021) 29(8):430–9. 10.12968/bjom.2021.29.8.430

[39] Hill M Scott J Cann H. The role of improvement during the response to COVID-19: insights from the Q community. London: The Health Foundation (2021). Available online at: https://q.thenhsalliance.org/assets/resources/The-role-of-improvement-during-the-response-to-COVID-19.pdf?v=1725287242 (Accessed July 2026)

