# Supplementary material for: Barriers and enablers to the implementation of a perinatal care bundle: the Perinatal Excellence to Reduce Injury in Premature Birth project
Source: Front Pediatr. 2026 Sep 16;14:1717781. doi: 10.3389/fped.2026.1717781 (PMC13624167; doi:10.3389/fped.2026.1717781)
Supplement: Supplementary file 1 [file Table1.docx]

**Part 1 - PERIPrem Bundle Component Questions**

1) Which components of the PERIPrem bundle are you focussing on at your trust?
2) Why did you choose to focus on these components?
3) Is there any of the components that you are not implementing at all? If so, why?
4) How do you feel the implementation of these components is going?
5) What are the difficulties you’ve experienced when implementing these components?

*[Probes: Can you tell more about….; How does that influence daily work?; Can you give examples...; What is your experience with...; Are there any other influencing factors you can think of?; How have you overcome these difficulties?]*

6) What has helped you to implement the components of the bundle?

*[Probes: Can you tell more about….; How does that influence daily work?; Can you give examples...; What is your experience with...; Are there any other influencing factors you can think of?]*

7) Have you made any adaptions to the components to help you with the implementation at your trust? If yes, please describe them.

*[Probes: Why did you make this adaption?; Can you think of the consequences of such an adaption being used?]*

**Part 2 – QI methodology Questions**

**You’re being supported to use QI methods to implement the bundle, through coaching, the share and learn sessions and additional resources.**

1) Tell me about your experiences with the coaching?

*[Probes: Is the support offered appropriate/ too much/ too little?]*

2) Have you attended any of the share and learn sessions? Were they useful? Why?
3) What do you think about the type of resources that have been made available? (i.e. video clips, Life QI)

*[Probes: Do you know how to access them?; Have you used them? How did you use them? Did you find them helpful?]*

4) What difficulties have you experienced implementing QI methodology?

*[Probes: Can you tell more about….; How does that influence daily work?; Can you give examples...; What is your experience with...; Are there any other influencing factors you can think of?]*

5) What has helped you to implement QI methodology?

*[Probes: Can you tell more about….; How does that influence daily work?; Can you give examples...; What is your experience with...; Are there any other influencing factors you can think of?]*

6) Have you had to make any adaptions to the QI methodology to help you with the implementation at your trust? If yes, please describe them.
7) Can you tell me about how the different teams are working together on the PERIPrem project?

*[Probes: Can you tell more about why you feel the teams are working well/ not working well?]*

8) Do you feel the project has helped bring the perinatal team together?
