# Supplementary material for: Barriers and enablers to the implementation of a perinatal care bundle: the Perinatal Excellence to Reduce Injury in Premature Birth project
Source: Front Pediatr. 2026 Sep 16;14:1717781. doi: 10.3389/fped.2026.1717781 (PMC13624167; doi:10.3389/fped.2026.1717781)
Supplement: Supplementary file 2 [file Table2.docx]

| **COM-B** | **TDF domain** | **TDF subdomain (did it act as a barrier and/or enabler)** |
| --- | --- | --- |
| Psychological Capability | Knowledge  *(awareness of the existence of something)* | Lack of procedural knowledge of bundle and QI (B/E) |
|  |  | Lack of awareness of the benefits of the bundle components (B/E) |
|  | Memory attention and decision processes  *(ability to retain information, focus selectively on aspects of the environment and choose between two or more alternatives)* | PERIPrem care bundle is complex and large (B) |
|  |  | The mental capacity of low, due to other demands (B) |
| Reflective motivation | Beliefs about consequences  *(acceptance of the truth, reality or validity about outcomes of a behaviour in a given situation)* | Strong, clear and relevant evidence for the interventions (B/E) |
|  |  | Credibility of the PERIPrem brand (E) |
|  | Goals  *(mental representations of outcomes or end states that an individual wants to achieve)* | PERIPrem had a clear goal and was shared by unit staff (E) |
| Physical Opportunity | Environmental context and resources  *(any circumstance of a person’s situation or environment that discourages or encourages the development of skills and abilities, independence, social competence and adaptive behaviour)* | Access to the PERIPrem resources and events (E) |
|  |  | Funding and time (B/E) |
|  |  | COVID-19 pandemic (B/E) |
|  |  | Size of unit (B/E) |
| Social Opportunity | Social influences  *(those interpersonal processes that can cause individuals to change their thoughts, feelings, or behaviours)* | Senior leadership, Trust and professional body endorsement (E) |
|  |  | Team collaboration and communication (B/E) |
|  |  | Co-production with staff (E) |
|  |  | PERIPrem leadership (B/E) |
|  |  | Change mindset at a team and trust level (E) |
