# Supplementary material for: Barriers and enablers to the implementation of a perinatal care bundle: the Perinatal Excellence to Reduce Injury in Premature Birth project
Source: Front Pediatr. 2026 Sep 16;14:1717781. doi: 10.3389/fped.2026.1717781 (PMC13624167; doi:10.3389/fped.2026.1717781)
Supplement: Supplementary file 3 [file Table3.docx]

|  |  | Page |
| --- | --- | --- |
| Domain 1: Research team and reflexivity | | |
| Personal Characteristics | | |
| 1. Interviewer/facilitator | Which author/s conducted the interview or focus group? | 4 |
| 2. Credentials | What were the researcher’s credentials? E.g., PhD, MD | 4 |
| 3. Occupation | What was their occupation at the time of the study? | 4 |
| 4. Gender | Was the researcher male or female? | 4 |
| 5. Experience and training | What experience or training did the researcher have? | 4 |
| Relationship with participants | | |
| 6. Relationship established | Was a relationship established prior to study commencement? | 4 |
| 7. Participant knowledge of the interviewer | What did the participants know about the researcher? e.g., personal goals, reasons for doing the research | 4 |
| 8. Interviewer characteristics | What characteristics were reported about the interviewer/facilitator? e.g., bias, assumptions, reasons, and interests in the research topic | 4 |
| Domain 2: study design | | |
| Theoretical framework | | |
| 9. Methodological orientation and Theory | What methodological orientation was stated to underpin the study? e.g., grounded theory, discourse analysis, ethnography, phenomenology, content analysis | 4/5 |
| Participant selection | | |
| 10. Sampling | How were participants selected? e.g., purposive, convenience, consecutive, snowball | 4 |
| 11. Method of approach | How were participants approached? e.g., face-to-face, telephone, mail, email | 4 |
| 12. Sample size | How many participants were in the study? | 5 |
| 13. Non-participation | How many people refused to participate or dropped out? Reasons? | 5 |
| Setting | | |
| 14. Setting of data collection | Where was the data collected? e.g., home, clinic, workplace | 4 |
| 15. Presence of non-participants | Was anyone else present besides the participants and researchers? | 4 |
| 16. Description of sample | What are the important characteristics of the sample? e.g., demographic data, date | 4 |
| Data collection | | |
| 17. Interview guide | Were questions, prompts, guides provided by the authors? Was it pilot tested? | Supplementary material |
| 18. Repeat interviews | Were repeat interviews carried out? If yes, how many? | 4 |
| 19. Audio/visual recording | Did the research use audio or visual recording to collect the data? | 4 |
| 20. Field notes | Were field notes made during and/or after the interview or focus group? | 4 |
| 21. Duration | What was the duration of the interviews or focus group? | 4 |
| 22. Data saturation | Was data saturation discussed? | n/a |
| 23. Transcripts returned | Were transcripts returned to participants for comment and/or correction? | 4 |
| Domain 3: analysis and findings | | |
| Data analysis | | |
| 24. Number of data coders | How many data coders coded the data? | 4/5 |
| 25. Description of the coding tree | Did authors provide a description of the coding tree? | 4/5 |
| 26. Derivation of themes | Were themes identified in advance or derived from the data? | 4/5 |
| 27. Software. | What software, if applicable, was used to manage the data? | 4/5 |
| 28. Participant checking | Did participants provide feedback on the findings? | 4/5 |
| Reporting | | |
| 29. Quotations presented | Were participant quotations presented to illustrate the themes / findings? Was each quotation identified? e.g., participant number | 5-10 |
| 30. Data and findings consistent | Was there consistency between the data presented and the findings? | 5-10 |
| 31. Clarity of major themes | Were major themes clearly presented in the findings? | 5-10 - barriers and enablers (themes) were presented in a clear structure, using the COM-B model. |
| 32. Clarity of minor themes | Is there a description of diverse cases or discussion of minor themes? | 5-10 – all barriers and enablers (themes) were reported, as were all behaviour change techniques |
